# Longitudinal relationships between social anhedonia and internalizing symptoms in autistic children: results from the Autism Biomarkers Consortium for Clinical Trials

**DOI:** 10.1017/S0033291725000650

**Published:** 2025-04-02

**Authors:** Alan H. Gerber, Adam Naples, Katarzyna Chawarska, Geraldine Dawson, Natalia Kleinhans, Shafali Jeste, Susan Faja, James Dziura, Sara Webb, Catherine Sugar, Frederick Shic, April R. Levin, James C. McPartland, for the Autism Biomarkers Consortium for Clinical Trials

**Affiliations:** 1 Child Study Center, Yale School of Medicine, New Haven, CT, USA; 2 Center for Autism, Children’s National Medical Center, Washington, DC, USA; 3 Department of Psychiatry and Behavioral Sciences, Duke University Medical Center, Durham, NC, USA; 4 Department of Radiology, University of Washington, Seattle, WA, USA; 5 Division of Neurology, Children’s Hospital Los Angeles, Los Angeles, CA, USA; 6 Division of Developmental Medicine, Boston Children’s Hospital, Boston, MA, USA; 7 Department of Biostatistics, Yale University, New Haven, CT, USA; 8 Seattle Children’s Research Institute, University of Washington, Seattle, WA, USA; 9 Department of Biostatistics, University of California Los Angeles, Los Angeles, CA, USA; 10 Department of Neurology, Boston Children’s Hospital, Boston, MA, USA

**Keywords:** autism spectrum disorders, children, depression, longitudinal, social anhedonia

## Abstract

**Background:**

Social anhedonia, indicating reduced pleasure from social interaction, is heightened in autistic youth and associated with increased internalizing symptoms transdiagnostically. The stability of social anhedonia over time and its longitudinal impact on internalizing symptoms in autism have never been examined.

**Methods:**

Participants were 276 autistic children (*M*_age_ = 8.60, SD_age_ = 1.65; 211 male) with IQ ≥ 60 (*M*_IQ_ = 96.74, SD_IQ_ = 18.19). Autism severity was measured using the Autism Diagnostic Observation Schedule, Second Edition. Caregivers completed the Child and Adolescent Symptom Inventory, Fifth Edition (CASI-5) at baseline, 6 weeks, and 6 months. The CASI-5 includes a social anhedonia subscale derived from relevant items across domains. ICC (Intraclass Correlation Coefficient) analysis assessed stability, while cross-lagged panel models examined associations among social anhedonia, depression, and social anxiety across time.

**Results:**

At baseline, social anhedonia correlated with autism severity, as well as parent-reported social anxiety and depression. Social anhedonia showed relative stability (ICC = 0.763) over 6 months, with a significant decline between baseline and 6 weeks (*β* = −0.52, *p* < .001). Cross-lagged models revealed a bidirectional relationship between social anhedonia and depression over time, while social anxiety displayed concurrent, but not predictive, associations across time.

**Conclusions:**

Social anhedonia demonstrated stability over 6 months, suggesting that it may be a relatively stable characteristic in autistic children. Concurrent relationships were observed between social anhedonia and depression, as well as social anxiety and attention-deficit/hyperactivity disorder. Only depression demonstrated a bidirectional longitudinal association with social anhedonia. This bidirectional relationship aligns with developmental models linking early negative social experiences to subsequent internalizing symptoms in autistic children, underscoring the clinical significance of social anhedonia assessment in this population.

## Introduction

Social anhedonia, reflecting reduced enjoyment from social interaction, is elevated in autistic youth (Chevallier et al., 2012). It is associated with higher internalizing symptoms, such as depression and anxiety (Gadow & Garman, 2020), in this population and accounts for greater variance in depression and social anxiety than autism symptoms (Gerber et al., 2025). Prior longitudinal research indicates that social anhedonia appears to be relatively stable among individuals with schizophrenia, while it is state-dependent in those experiencing depression (Blanchard, Horan, & Brown, 2001). However, no longitudinal work has been conducted with autistic children. Thus, whether social anhedonia in autistic children is stable across time – and how this may impact internalizing symptoms – remains unknown.

## Social anhedonia as a transdiagnostic factor

Social interaction is one of the most basic and fundamental human needs (Baumeister & Leary, 1995; Van Lange & Columbus, 2021), serving as protection against physical and mental health risks associated with isolation and loneliness (Holt-Lunstad et al., 2015; Holt-Lunstad, Smith, & Layton, 2010; House, Landis, & Umberson, 1988). Social anhedonia is a clinical characteristic defined by a reduction in pleasure from interpersonal interaction (Meehl, 1975; Myerson, 1922; Ribot, 1897). It is an important transdiagnostic marker implicated in a wide range of psychiatric and neurodevelopmental conditions, including autism, depression, schizophrenia, and eating disorders (Barkus & Badcock, 2019; Gandhi, Mote, & Fulford, 2022). Symptoms of social anhedonia are linked with altered neural response to social reward (Healey et al., 2014; Wang, Li, Nie, & Zheng, 2020; Zhang et al., 2020) and have been associated with increased risk for mood disorders and suicidality in clinical populations (Blanchard et al., 2009; Harrison, Mountford, & Tchanturia, 2014; Sagud et al., 2021; Tchanturia et al., 2012; Yang et al., 2020).

Social anhedonia in the general population increases in prevalence from childhood into adolescence (Dodell-Feder & Germine, 2018; Yang, Guo, Harrison, & Liu, 2022). In individuals with schizophrenia, symptoms of social anhedonia remain relatively stable despite symptom improvement, indicating that it may be an ongoing characteristic in these individuals (Blanchard, Horan, & Brown, 2001). In contrast, depressed individuals who experience symptom improvement report decreased social anhedonia (Blanchard, Horan, & Brown, 2001). This is consistent with a recent meta-analysis indicating differences in the stability of social anhedonia between schizophrenia and depression (Gandhi, Mote, & Fulford, 2022). Overall, while social anhedonia is a transdiagnostic construct, it is possible that the mechanism underlying the development and maintenance of social anhedonia may vary across psychiatric conditions.

## Social anhedonia in autistic children

Early characterizations of autism posited that social anhedonia was a hallmark characteristic of autism (Kanner, 1943), although it has not been defined as a core diagnostic feature. On average, autistic adolescents and adults report higher symptoms of social anhedonia compared with non-autistic individuals (Berthoz, Lalanne, Crane, & Hill, 2013; Carré et al., 2015; Chevallier et al., 2012; Han, Tomarken, & Gotham, 2019). Parents also report elevations in social anhedonia in autistic children as young as age 6 (Gadow & Garman, 2020; Gerber et al., 2025). However, interests in social interaction are known to vary across autistic individuals with some showing a high interest in interacting with others (e.g. Meng et al., 2018; Neuhaus, Bernier, & Webb, 2021; Scheeren, Koot, & Begeer, 2020; Zhao et al., 2024). Symptoms of social anhedonia in autistic children are associated with heightened internalizing symptoms, such as depression and anxiety (Gadow & Garman, 2020), and account for greater variance in depression and social anxiety than autism symptoms (Gerber et al., 2025), highlighting its clinical importance. In contrast, symptoms of attention-deficit/hyperactivity disorder (ADHD) appear to be negatively correlated with social anhedonia in autistic youth (Gerber et al., 2025). Overall, social anhedonia represents an important transdiagnostic clinical characteristic for understanding symptom presentation and with potential relevance to clinical decision-making in autistic children.

Given its concurrent relationship with internalizing symptoms, it is important to understand whether social anhedonia has predictive clinical value in autism. However, no work has explored the variability of social anhedonia across time or the longitudinal relationships between social anhedonia and internalizing symptoms in autistic children. Theoretical models of the development of internalizing symptoms in autistic children posit bidirectional relationships between social withdrawal and internalizing symptoms over time (Wood & Gadow, 2010; Yarger & Redcay, 2020). Early symptoms of social anhedonia could lead to social withdrawal and thus represent an important link to an increased risk of internalizing symptoms. Therefore, understanding the role of social anhedonia in the developmental course of internalizing symptoms in autistic children is essential for understanding relevant timing for potential therapeutic support.

## Current study

This study utilized data obtained from a well-characterized sample of autistic children and their caregivers during in-person research visits across the course of 6 months. The objective of the current set of analyses was to assess the concurrent relationships between social anhedonia and co-occurring psychiatric symptoms, estimate the 6-month stability of social anhedonia in autistic children, and examine its longitudinal relationship with internalizing symptoms. Analyses were centered on the most common co-occurring psychiatric conditions in autistic children, including anxiety, depression, and ADHD (Kerns, Rast, & Shattuck, 2020; Simonoff et al., 2008).

We hypothesized that social anhedonia symptoms would be significantly associated with co-occurring psychiatric symptoms in autistic children after controlling for autism symptoms, age, sex, and intelligence quotient (IQ) (Hypothesis 1). Based on prior work, we specifically hypothesized that this would be the case for symptoms of depression, social anxiety, and ADHD (Gerber et al., 2025). We also examined the trajectory of social anhedonia symptoms in autistic children over the course of 6 months and whether this trajectory was moderated by age, sex, or IQ (Hypothesis 2). Lastly, we hypothesized that social anhedonia would explain unique variance in the development of co-occurring psychiatric symptoms, specifically depression and social anxiety, after accounting for autoregressive and concurrent relationships (Hypothesis 3).

## Methods

### Participants

Participants in this study were autistic children drawn from a larger study examining biomarkers in autism, the Autism Biomarkers Consortium for Clinical Trials (ABC-CT; McPartland et al., 2020). All participants were enrolled in the study between the ages of 6 and 11. Autistic children were required to meet the criteria for autism on standardized diagnostic assessments and have a composite IQ score between 60 and 150. Exclusion criteria included known genetic conditions and significant visual, auditory, or motor challenges that would limit study participation. The final sample included 276 autistic children between the ages of 6 and 11 (*M* = 8.60 years, SD = 1.65). See Table 1 for the sample demographics.

**Table 1.** Demographic characteristics of the sample

Variables	*N* = 276
Age, mean (SD)	8.60 (1.65)
Sex, *n* (%)	
Female	65 (24%)
Male	211 (76%)
Ethnicity, *n* (%)	
Hispanic or Latino	52 (19%)
Non-Hispanic or Latino	224 (81%)
Race, *n* (%)	
American Indian/Alaskan Native	2 (0.7%)
Asian	15 (5.4%)
Black or African American	21 (7.6%)
White	188 (68%)
More than one race	44 (16%)
Other	6 (2.2%)
Family income, *n* (%)	
≤$35,000	37 (14%)
$35,001–$50,000	24 (8.9%)
$50,001–$75,000	32 (12%)
$75,001–$100,000	38 (14%)
$100,001–$150,000	64 (24%)
>$150,000	74 (27%)
Unsure	1 (0.4%)

### Procedure

Participants for ABC-CT were recruited from five research centers across the United States (Boston Children’s Hospital; Duke University; University of California, Los Angeles; University of Washington; and Yale University). Children completed diagnostic and cognitive assessments during in-person site visits. Their parents and caregivers completed paper questionnaires sent out in advance and collected during the in-person visit. Data were collected at three different timepoints, baseline, 6 weeks after baseline, and 6 months after baseline. Families were given a 2-week window before and after both follow-up visits to complete data collection. Clinicians were assigned to specific families and consistently administered diagnostic interviews for that family across timepoints whenever possible. Autism diagnosis required meeting criteria on gold-standard diagnostic assessments in addition to clinical judgment based on the DSM-5 criteria. Study procedures were approved by the Yale Institutional Review Board (IRB), which operated as a central IRB for this study. All parents and caregivers provided informed consent for study participation.

### Measures

#### Autism diagnostic status and symptom severity

The Autism Diagnostic Observation Schedule, Second Edition (ADOS-2; Lord et al., 2012) is a standardized diagnostic tool that was used to confirm autism diagnosis for all participants. It consists of a semistructured set of social tasks designed to elicit social behaviors as well as any present restrictive and repetitive behaviors. The ADOS-2 yields a diagnostic classification and calibrated severity score, which can be compared across modules. Most participants completed Module 3 intended for youth with fluent speech (230; 83.3%); however, 40 participants completed Module 2 (14.5%) and 6 participants completed Module 1 (2.2%). Research reliable clinicians completed all ADOS-2 assessments, and 10% of recorded videos were co-scored for ongoing evaluation of reliability.

The Autism Diagnostic Interview–Revised (ADI-R; Lord, Rutter, & Le Couteur, 1994) is a gold-standard, structured diagnostic parent interview designed to assess historical and current behaviors related to an autism diagnosis. It provides detailed information across three core diagnostic domains: Language/Communication, Reciprocal Social Interactions, and Repetitive Behaviors/Interests. All ADI-R interviews in this study were administered by research-reliable clinicians.

The Social Responsiveness Scales, Second Edition (SRS-2; Constantino & Gruber, 2012) is a parent-report questionnaire measuring autism symptom severity. It consists of 65 items rated on a 4-point Likert scale. The SRS-2 provides a sex-normed composite T-score representing overall symptom severity, with higher T-scores reflecting greater symptom severity. All caregivers completed the school-age SRS-2 form. Reliability for the SRS-2 at baseline in the data was excellent (Cronbach’s *α* = .94).

#### Cognitive assessment

Cognitive ability and information processing were assessed using the Differential Ability Scales-II (DAS-II; Elliott, 2007). The DAS-II is a clinician-administered assessment that produces a verbal and nonverbal IQ, as well as an age-normed standardized IQ score. All participants completed the DAS-II school-age version core battery.

#### Co-occurring psychiatric symptoms

Co-occurring psychiatric symptoms were assessed with the Child and Adolescent Symptom Inventory, Fifth Edition (CASI-5; Gadow & Sprafkin, 2012). The CASI-5 is a 173-item parent-report questionnaire in which parents are asked to rate their children’s current behavior on a 4-point Likert scale from *never* to *very often.* It has been widely used to assess symptoms of autism and co-occurring psychiatric conditions in autistic youth (e.g. Brenner et al., 2018; Duan et al., 2022; Plesa Skwerer et al., 2019). Consistent with our prior work, this study utilized the subscales measuring ADHD, social anxiety, generalized anxiety, separation anxiety, and depression symptoms (Gerber et al., 2025). The CASI-5 produces a T-score for each condition that is normed by age and sex. The ABC-CT study included 280 autistic children; however, four participants were missing CASI-5 data at baseline and were therefore excluded from further analyses. Reliability at baseline for each of the five CASI-5 subscales utilized for this study was good (all Cronbach’s *α* > .80).

#### Social anhedonia

Social anhedonia symptoms were measured using a set of six items drawn from the depression, schizoid personality, and autism subscales of the CASI-5. The subscale includes one item out of the seven from the depression subscale (*shows little interest in or enjoyment of pleasurable activities*). It also includes all three items from the schizoid personality subscale (*prefers to be alone rather than with friends or family; shows little interest in having close relationships; is emotionally cold or indifferent to people*) and 2 of the 16 items from the autism subscale (*not interested in making friends; is unaware or takes no interest in other people’s feelings*). Items from the social anxiety subscale and those related to social skills were specifically excluded from this subscale. The chosen items reflect observable symptoms of social anhedonia and contain content consistent with typical measures of social anhedonia, such as reduced interest in close relationships and a preference for being alone (see Gadow & Garman, 2020). These six items were summed to create a total social anhedonia symptom score as per Gadow and Garman (2020), with higher total scores indicating greater symptom severity. Following cutoff procedures defined by Gadow and Garman (2020), when caregivers endorsed two or more symptoms of social anhedonia (i.e. rated them as *often* or *very often*), children were classified as being above the cutoff for social anhedonia, while children with no symptoms were classified as not meeting criteria for social anhedonia. According to scoring procedures established by Gadow and Garman (2020), children whose caregivers endorsed only one symptom did not receive a classification in order to increase differentiation across groups. Therefore, these children were not included when presenting the youth meeting the cutoff for social anhedonia. However, all other analyses used a dimensional approach utilizing the continuous social anhedonia total score. This subscale has been previously used to measure social anhedonia in autistic youth (Gadow & Garman, 2020; Gerber et al., 2025). Reliability for the social anhedonia subscale at baseline was good (Cronbach’s α = .80).

### Data analytic plan

Means and standard deviations for key demographic and clinical variables were computed. Next, first-order correlations at baseline were calculated between each of these key variables. All analyses were completed in R version 4.3.2 (R Core Team, 2024). To examine levels of social anhedonia in autistic children, we ran further descriptive analysis regarding the total number of children meeting criteria and the distribution of social anhedonia scores across time. The relationships between social anhedonia with age and sex were also investigated.

To test whether social anhedonia symptoms had a significant association with key co-occurring psychiatric symptoms after controlling for autism symptoms, age, sex, and IQ (Hypothesis 1), a series of five separate multiple regressions were run. In each of the regression models, autism symptoms, age, sex, and IQ were entered as the predictors, while the dependent variable was one of the five key co-occurring psychiatric symptoms.

A multilevel modeling (MLM) framework was used to examine trajectories of social anhedonia symptoms across time (Hypothesis 2). MLM analyses were run using the lme4 package in R (Bates, Mächler, Bolker, & Walker, 2015) and a Full Maximum Likelihood estimation procedure. Models were compared using log-likelihood ratio tests. All continuous predictors (i.e. age and IQ) were standardized. Time was modeled as an ordinal variable with three timepoints (baseline, 6 weeks, and 6 months). First, the need for random effects was tested, with future models retaining those effects. The next timepoint was added to the model predicting social anhedonia symptoms. Finally, models including the effects of age, sex, and IQ on trajectories of social anhedonia symptoms were tested.

Structural equation models were fit to the data to examine the longitudinal relationship between social anhedonia and co-occurring psychiatric symptoms. Specifically, cross-lagged panel models were used to estimate the autoregressive, concurrent, and cross-lagged relationships between social anhedonia and co-occurring psychiatric symptoms. Importantly, by accounting for concurrent relationships, cross-lagged coefficients in the model are independent of these associations and specifically represent the directional influence of variables over time. Only co-occurring conditions that were associated with social anhedonia at baseline after controlling for autism symptoms were investigated. Each co-occurring psychiatric symptom was examined in a separate model. All cross-lagged panel analyses were completed with the Lavaan package in R (Rosseel, 2012). Power analysis was conducted using the semPower package in R (Moshagen & Bader, 2024). Assuming autocorrelation coefficients of .8 across waves, a sample size of 276 was sufficient for detecting a cross-lagged effect size of .1 with a power of .8.

## Results

Means and standard deviations for key variables, as well as zero-order correlations, are presented in Table 2. Social anhedonia total scores were associated with clinician-rated autism severity on the ADOS-2, as well as most parent-report variables. ADOS-2 symptom severity scores were also related to lower IQ scores, CASI-5 ADHD T-scores, CASI-5 generalized anxiety T-scores, and being male.

**Table 2.** Means, standard deviations, and correlations of demographic and clinical variables

Variables	M	SD	1	2	3	4	5	6	7	8	9	10
1. Age	8.60	1.65										
2. Male	0.76	0.43	0.08									
3. ADOS–2 CSS	7.63	1.77	−0.10	0.22^**^								
4. Composite IQ	96.74	18.19	0.09	−0.14*	−0.29^**^							
5. SRS–2 Total T-Score	73.56	10.94	0.05	−0.12*	0.05	−0.11						
6. CASI–5 ADHD T-Score	73.69	13.08	−0.03	−0.26^**^	−0.12*	0.12*	0.56^**^					
7. CASI–5 Social Anxiety T-Score	61.10	17.30	−0.01	−0.17^**^	0.06	−0.02	0.42^**^	0.08				
8. CASI–5 Generalized Anxiety T-Score	68.45	16.07	0.21^**^	−0.21^**^	−0.20^**^	0.15*	0.49^**^	0.53^**^	0.31^**^			
9. CASI–5 Separation Anxiety T-Score	56.16	14.87	−0.03	−0.15*	−0.05	−0.03	0.27^**^	0.17^**^	0.38^**^	0.44^**^		
10. CASI–5 Depression T-Score	59.09	16.77	0.15*	−0.04	−0.11	0.09	0.43^**^	0.31^**^	0.31^**^	0.71^**^	0.46^**^	
11. CASI–5 Social Anhedonia Total	4.59	3.51	0.10	0.06	0.13*	−0.04	0.60^**^	0.19^**^	0.37^**^	0.28^**^	0.21^**^	0.39^**^

*Note*: ADOS-2 CSS, Autism Diagnostic Observation Schedule, Second Edition Calibrated Severity Score; CASI-5, Child and Adolescent Symptom Inventory, Fifth Edition; SRS-2, Social Responsiveness Scale, Second Edition.

* *p* < 0.05.

** *p* < 0.01.

### Social anhedonia in autistic children

**Figure 1.**
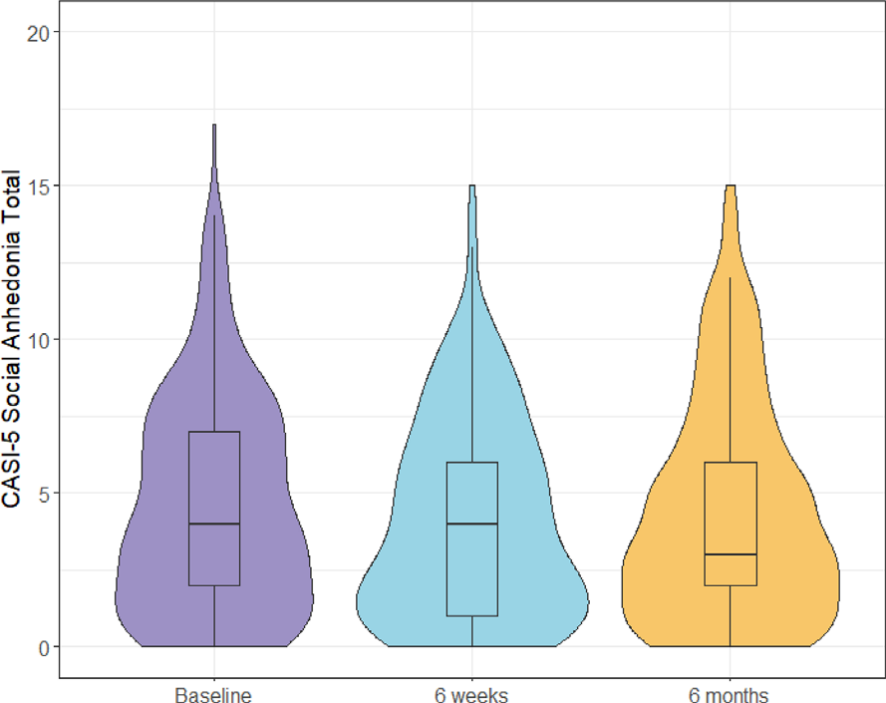
Distribution of Child and Adolescent Symptom Inventory, Fifth Edition social anhedonia scores for autistic children across all three timepoints of the study.

Of the 276 autistic children in the sample, 52 children had a parent report endorsing only one symptom of social anhedonia and were not provided a qualitative classification (see the Methods section for details). There were 84 autistic children out of the remaining 224 that met the criteria for social anhedonia (38%). The distribution of social anhedonia scores for autistic children is shown in Figure 1. Social anhedonia was not associated with the participant age (*b* = 0.21, SE = 0.13, *p* = 0.103) or sex (*t*(120.59) = 1.16, *p* = .250). Further, we found no differences in social anhedonia across participant ethnicity (*t*(77.48) = 0.85, *p* = .400), participant race (*F*(5,270) = 0.89, *p* = .488), or family income (*F*(5,270) = 1.24, *p* = .287).

### Social anhedonia and co-occurring symptoms

Results of linear regression models predicting co-occurring psychiatric symptoms are displayed in Table 3. All overall regression models were significant, and greater autism symptoms were associated with higher symptoms for all co-occurring psychiatric conditions. Greater social anhedonia symptoms were associated with higher symptoms of social anxiety and depression after accounting for autism symptoms. Further, greater social anhedonia symptoms were related to lower ADHD symptoms after accounting for autism symptoms.

**Table 3.** Results of linear regression models predicting co-occurring psychiatric symptoms

Predictors	ADHD		Social anxiety		Generalized anxiety		Separation anxiety		Depression	
	*β* (SE)	*p*	*β* (SE)	*p*	*β* (SE)	*p*	*β* (SE)	*p*	*β* (SE)	*p*
Intercept	11.68 (7.13)	0.103	31.88 (10.72)	**0.003**	−10.31 (9.30)	0.269	43.54 (9.99)	**<0.001**	−1.10 (10.33)	0.915
Autism symptoms	0.82 (0.07)	**<0.001**	0.43 (0.11)	**<0.001**	0.72 (0.09)	**<0.001**	0.26 (0.10)	**0.011**	0.49 (0.11)	**<0.001**
Social anhedonia symptoms	−0.73 (0.22)	**0.001**	1.11 (0.33)	**0.001**	−0.06 (0.29)	0.847	0.46 (0.31)	0.146	0.94 (0.32)	**0.004**
Age	−0.37 (0.38)	0.333	−0.41 (0.57)	0.470	1.80 (0.49)	**<0.001**	−0.36 (0.53)	0.500	1.07 (0.55)	0.051
IQ	0.12 (0.03)	**<0.001**	0.00 (0.05)	0.934	0.15 (0.05)	**0.001**	−0.02 (0.05)	0.689	0.12 (0.05)	**0.022**
Sex (male)	−4.53 (1.51)	**0.003**	−5.55 (2.28)	**0.015**	−5.30 (1.97)	**0.008**	−4.82 (2.12)	**0.024**	−0.37 (2.19)	0.865
*R* ^2^	0.405		0.222		0.330		0.098		0.240	

*Note*: Regression coefficients (*β*) with standard errors (SE). Significant predictors are in bold. The reference group for beta coefficients is in parentheses. ADHD, attention-deficit/hyperactivity disorder.

### Trajectories of social anhedonia across 6 months

Overall, missing data were limited, and attrition rates were low. Of the 276 participants with baseline data, 271 (98.19%) had CASI-5 data at a 6-week follow-up and 257 (93.16%) had CASI-5 data at a 6-month follow-up.

The overall ICC for the model of time predicting changes in social anhedonia was .77 (see model 1 of Table 4). This indicates that 77% of the variance in social anhedonia in the model was associated with between-person differences, suggesting that social anhedonia symptoms were relatively stable at the within-person level. Figure 1 displays the distribution of social anhedonia total scores at each timepoint. There was a significant decline in social anhedonia symptoms between baseline and 6-week follow-up (*B* = −0.52, SE = 0.14, *p* < .001); however, there was no difference between baseline and 6-month follow-up (see model 1 of Table 4). The trajectory of social anhedonia symptoms over time was not impacted by age, sex, or IQ (see models 2–4 of Table 4).

**Table 4.** Results of multilevel models predicting social anhedonia symptoms over 6 months

Predictors	Model 1 (time)	Model 2 (age)	Model 3 (sex)	Model 4 (IQ)
	*β* (SE)	*β* (SE)	*β* (SE)	*β* (SE)
Intercept	4.59 (0.21)^***^	4.59 (0.20)^***^	4.18 (0.42)^***^	4.59 (0.21)^***^
6 Weeks	−0.52 (0.14)^***^	−0.52 (0.14)^***^	−0.60 (0.29)*	−0.52 (0.14)^***^
6 Months	−0.23 (0.14)	−0.23 (0.14)	−0.05 (0.30)	−0.23 (0.14)
Age		0.35 (0.20)		
Age × 6 Weeks		−0.10 (0.14)		
Age × 6 Months		−0.03 (0.14)		
Sex (male)			0.54 (0.48)	
Sex × 6 Weeks			0.11 (0.33)	
Sex (male) × 6 Months			−0.23 (0.34)	
IQ				−0.13 (0.21)
IQ × 6 Weeks				0.03 (0.14)
IQ × 6 Months				0.07 (0.14)
*Random effects*				
*σ* ^2^	2.70	2.70	2.70	2.70
*τ*_00_	8.94	8.86	8.91	8.94
ICC	0.77	0.77	0.77	0.77
Marginal *R* ^2^/conditional *R* ^2^	0.004/0.769	0.012/0.769	0.008/0.770	0.005/0.769

*Note:* Standardized regression coefficients (*β*) with standard errors (SE). The reference group for beta coefficients is in parentheses.

* *p* < 0.05.

** *p* < 0.01.

*** *p* < 0.001.

### Longitudinal relationships between social anhedonia and co-occurring symptoms

**Figure 2.**
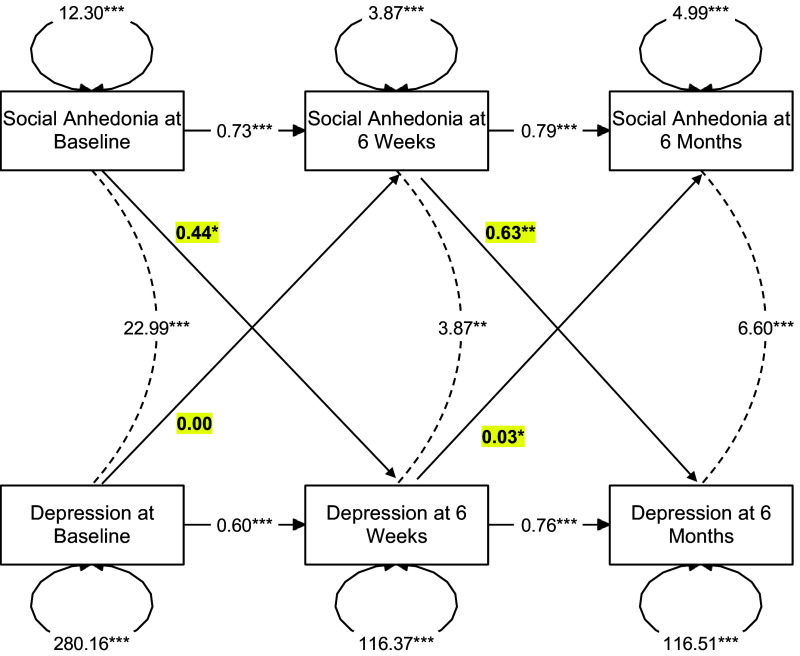
Results of a cross-lagged panel model representing the autoregressive, concurrent, and cross-lagged relationships between social anhedonia and depression. *Note*: Cross-lagged effects are bolded and highlighted in yellow. Social anhedonia and depression are concurrently related to each other at all three timepoints. In addition, they demonstrate a bidirectional relationship over time, such that each is significantly related to the other at future timepoints, with the exception of baseline depression and social anhedonia at 6 weeks.

**Figure 3.**
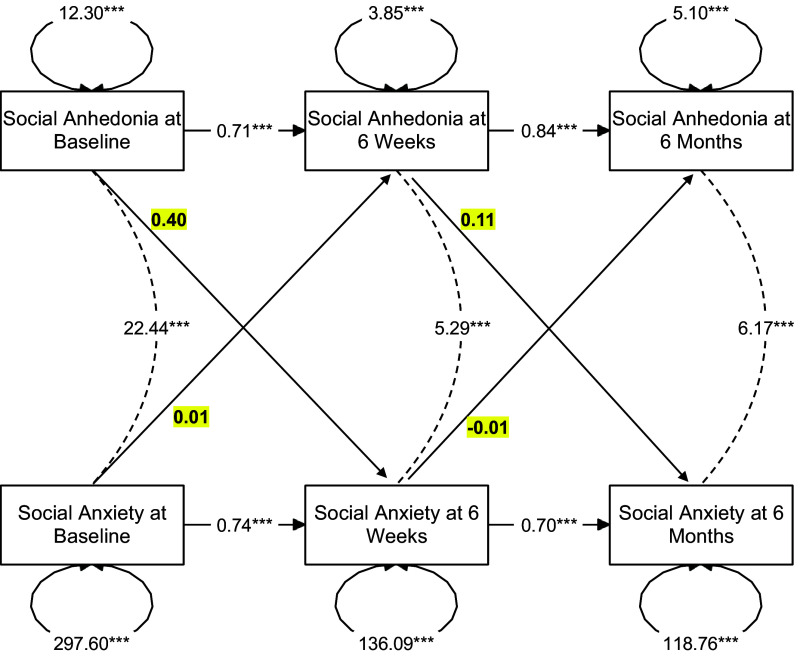
Results of a cross-lagged panel model representing the autoregressive, concurrent, and cross-lagged relationships between social anhedonia and social anxiety. *Note*: Cross-lagged effects are bolded and highlighted in yellow. Social anhedonia and social anxiety are concurrently related to each other at each timepoint; however, they do not significantly predict each other over time.

**Figure 4.**
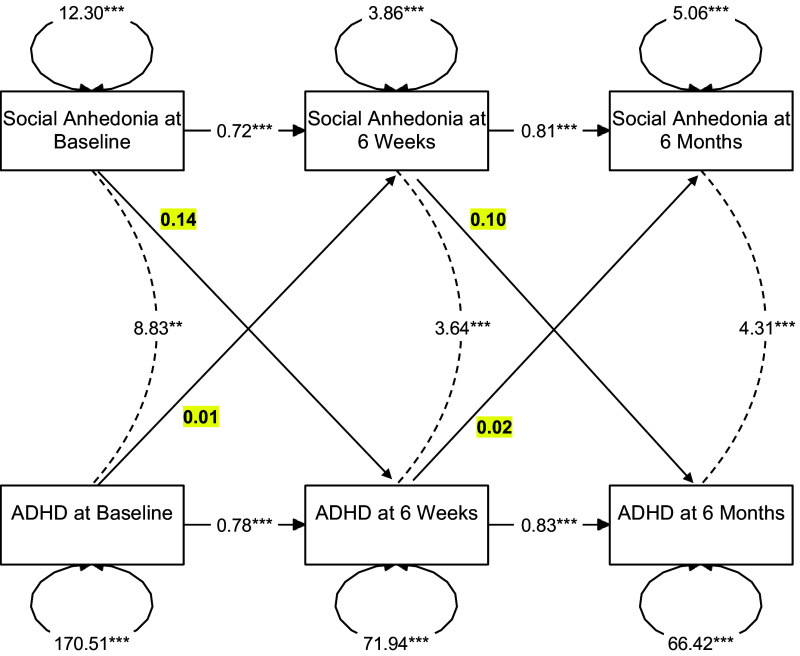
Results of a cross-lagged panel model representing the autoregressive, concurrent, and cross-lagged relationships between social anhedonia and attention-deficit/hyperactivity disorder (ADHD). *Note*: Cross-lagged effects are bolded and highlighted in yellow. Social anhedonia and ADHD are concurrently related to each other at each timepoint; however, they do not significantly predict each other over time.

Standardized parameter estimates for each of the cross-lagged panel models are displayed in Figures 2– 4. Symptoms of social anhedonia consistently demonstrated significant autoregressive effects between prior timepoints across all models (all *β*s > 0.70, all *p*s < 0.001). In the model examining the relationship between social anhedonia and depression (Figure 2), there were significant autoregressive effects found between depression symptoms across all timepoints (all *β*s > 0.59, all *p*s < 0.001). There was also a concurrent positive relationship between social anhedonia and depression symptoms at baseline (cov = 22.99, SE = 3.80, *p* < 0.001), 6-week follow-up (cov = 3.87, SE = 1.31, *p* = 0.003), and 6-month follow-up (cov = 6.60, SE = 1.56, *p* < 0.001). Finally, there was a significant cross-lagged relationship between social anhedonia symptoms at baseline and depression symptoms at 6-week follow-up (*β* = 0.44, SE = 0.20, *p* = 0.029), as well as social anhedonia symptoms at 6-week follow-up and depression symptoms at 6-month follow-up (*β* = 0.63, SE = 0.22, *p* = 0.005), such that social anhedonia predicted increased depression. There was also a cross-lagged relationship between depression symptoms at 6-week follow-up and social anhedonia symptoms at 6-month follow-up (*β* = 0.03, SE = 0.01, *p* = 0.012), such that depression predicted increased social anhedonia. However, the cross-lagged relationship between depression symptoms at baseline and social anhedonia symptoms at 6-week follow-up (*β* = 0.00, SE = 0.01, *p* = 0.953) was not significant.

In the model examining the relationship between social anhedonia and social anxiety (Figure 3), there were significant autoregressive effects found between social anxiety symptoms across all timepoints (all *β*s > 0.69, all *p*s < 0.001). There was also a concurrent positive relationship between social anhedonia and social anxiety symptoms at baseline (cov = 22.44, SE = 3.88, *p* < 0.001), 6-week follow-up (cov = 5.30, SE = 1.43, *p* < 0.001), and 6-month follow-up (cov = 6.17, SE = 1.59, *p* < 0.001). Finally, there were no significant cross-lagged relationships between social anhedonia symptoms and social anxiety symptoms (all *β*s < 0.40, all *p*s > 0.067).

Lastly, in the model examining the relationship between social anhedonia and ADHD (Figure 4), there were significant autoregressive effects found between ADHD symptoms across all timepoints (all *β*s > 0.77, all *p*s < 0.001). There was also a concurrent positive relationship between social anhedonia and ADHD symptoms at baseline (cov = 8.83, SE = 2.81, *p* = 0.002), 6-week follow-up (cov = 3.64, SE = 1.04, *p* < 0.001), and 6-month follow-up (cov = 4.31, SE = 1.18, *p* < 0.001). Finally, there were no significant cross-lagged relationships between social anhedonia symptoms and ADHD symptoms (all *β*s < 0.14, all *p*s > 0.100).

## Discussion

To the best of our knowledge, this was the first study to examine longitudinal trajectories of social anhedonia and their clinical correlates in autistic children. Consistent with our first hypothesis, we found that social anhedonia symptoms were concurrently associated with internalizing symptoms at baseline in autistic children even after controlling for autism severity. Notably, symptoms of social anhedonia were similar across age, sex, race/ethnicity, and family income, underscoring its clinical utility in autistic children. We also found a relatively stable trajectory of social anhedonia symptoms in autistic children that remained consistent across demographic characteristics. Finally, we found that symptoms of social anhedonia predicted increases in depression; this was not the case for symptoms of social anxiety or ADHD. Overall, these results converge with existing research highlighting the clinical importance of social anhedonia in autistic children.

### Social anhedonia in autistic children

Congruent with previous research, autistic children varied in their degree of interest in social interaction, with a little over one-third of the sample (38%) meeting the criteria for social anhedonia on parent reports. This is in line with the literature indicating elevated symptoms of social anhedonia in this population (Chevallier et al., 2012; Gadow & Garman, 2020). However, these levels are below prior reports, which found that about half of autistic children met the criteria for social anhedonia (Gerber et al., 2025). Given that symptoms of anhedonia in children generally peak around adolescence (Dodell-Feder & Germine, 2018; Gupta, Eckstrand, & Forbes, 2024), it is likely that differences in sample age contributed to this finding. Specifically, the sample analyzed by Gerber et al. (2025) included children aged 8–18, while the current study examined a sample of children aged 6–11. This difference in sample age may also explain why the study by Gerber et al. (2025) found an association between social anhedonia and age when the current study did not. Nonetheless, we still found significant levels of social anhedonia in this study, suggesting that it is important to assess in younger autistic children.

Although our findings suggest a longitudinal relationship between social anhedonia and depression, depression rates are generally higher in female autistic adolescents compared with their male counterparts (Oswald et al., 2016; Schwartzman, Williams, & Corbett, 2022). Thus, it is noteworthy that we did not find comparable sex differences in social anhedonia. However, these findings are consistent with prior work (Gadow & Garman, 2020; Gerber et al., 2025), suggesting that there may be elements of multifinality, such that symptoms of social anhedonia may exhibit differential relationships with co-occurring psychiatric symptoms in autistic males and females. Alternatively, it is possible that the diagnostic criteria for depression may be less attuned to identifying depression in autistic males compared with autistic females, potentially leading to underdiagnosis in males despite similar clinical profiles (Zheng et al., 2021). Taken together, these findings highlight the need for further research to explore sex differences in co-occurring psychiatric conditions and their relationship to social anhedonia in autistic youth.

Further, our results demonstrate a cross-sectional relationship between symptoms of social anhedonia and internalizing symptoms above and beyond autism symptom severity. Specifically, we found that symptoms of social anxiety and depression were concurrently associated with greater social anhedonia, while generalized and separation anxiety were not. Overall, these findings replicate prior research (Gerber et al., 2025) in an independent sample and extend them to younger autistic children. Further, the results indicated that symptoms of ADHD were linked to lower social anhedonia, consistent with our prior findings (Gerber et al., 2025). Previous work has shown that youth with ADHD exhibit heightened responsiveness to social rewards (Kohls, Herpertz-Dahlmann, & Konrad, 2009), while co-occurring ADHD in autistic youth has been linked to a relatively heightened responsiveness to social rewards (Baumeister et al., 2023). Thus, in the context of the broader literature, our findings suggest the possibility of a clinically relevant autistic phenotype characterized by hyper- rather than hypo-responsiveness to social rewards (e.g. Yi, Wang, Song, & Han, 2022).

### Trajectories of social anhedonia

Results indicated that social anhedonia was consistent across 6 months in autistic youth. This suggests that it may be a relatively stable characteristic in autistic children, consistent with social anhedonia symptoms for those on the schizophrenia spectrum rather than the state-like changes seen in individuals with depression (Blanchard, Horan, & Brown, 2001). However, it is also possible that 6 months may not provide enough time to effectively assess changes in social anhedonia in autistic youth. Thus, future research should examine longitudinal stability across longer periods of time. Further, additional research is needed to better understand how stable social anhedonia is across the lifespan in autism and whether specific factors contribute to variability in this characteristic over time. In the present study, there was a significant group-level decrease in social anhedonia between baseline and 6-week follow-up. It is possible that improvements in social anhedonia symptoms at 6 weeks could be a result of the sustained contact involved in participating in a longitudinal study. This suggests that external factors can influence the presence of social anhedonia in autism. Nonetheless, trajectories of social anhedonia were not different for children based on age, sex, and IQ. Thus, symptoms of social anhedonia may represent an important transdiagnostic clinical characteristic and potential intervention target in autistic children.

Further, the current study presents evidence regarding the longitudinal associations between symptoms of social anhedonia and internalizing symptoms in autistic children. Contrary to our hypotheses, our investigation did not reveal a significant predictive relationship between symptoms of social anhedonia and social anxiety over the 6-month study period. However, despite the relative stability of social anhedonia in autistic youth, findings demonstrated a bidirectional relationship between symptoms of social anhedonia and depression over time. This suggests a reciprocal influence between social anhedonia and depression, where these constructs iteratively impact each other over time. These results build upon prior research, underscoring the pivotal role of social anhedonia symptoms as a risk factor for depression in autistic children. Our findings are consistent with developmental models of internalizing symptoms in autistic children, which propose that early negative social experiences lead to social withdrawal and downstream internalizing symptoms (Wood & Gadow, 2010; Yarger & Redcay, 2020). Overall, this highlights the critical importance of assessing symptoms of social anhedonia in autistic children and lays the groundwork for targeted interventions for autistic children.

### Limitations

This study is subject to several limitations that warrant consideration. First, while the CASI-5 social anhedonia subscale has demonstrated strong internal reliability in prior use (Gadow & Garman, 2020; Gerber et al., 2025), it nonetheless has limited data in terms of psychometric validation. This limitation exacerbates challenges with overlapping items on the depression and autism subscales, restricting conclusions regarding the specificity of these results. Further, reliance on parent report introduces potential difficulties in discriminating among social anhedonia, depression, and social anxiety, given the behavioral overlap between these constructs. For example, without an understanding of the internal context, parents may conflate symptoms of social anhedonia (lack of desire for social contact) with symptoms of social anxiety (difficulty engaging in social contact). This may be particularly relevant for the youth with limited language who completed an ADOS module 1 or 2, where it may be less clear what constitutes social anhedonia. Additionally, although we examined trajectories of social anhedonia over a 6-month period, this time frame may not be sufficient to capture measurable changes. Further, it is possible that some participants received interventions during the course of this longitudinal study, which may have influenced our findings. Finally, this study did not include a comparison group of neurotypical youth, limiting the ability to assess the specificity of these effects. Future research should address this gap by examining whether these findings are unique to autistic children.

### Conclusion

In summary, this work replicates prior work indicating elevated prevalence of social anhedonia in autistic children and the concurrent relationships between these symptoms and co-occurring psychopathology. Our findings also shed light on the trajectories of social anhedonia in autistic children, revealing a relatively stable trajectory over the course of 6 months. Finally, the results demonstrate a bidirectional relationship between social anhedonia and depression symptoms over time, suggesting that symptoms of social anhedonia may help identify autistic children at risk for the development of depression. Future research should integrate objective indicators of social reward processing (e.g. neural or visual attentional biomarkers) with questionnaire and parent-report measures to improve understanding of social anhedonia in autistic youth. Overall, these results underscore the clinical significance of identifying autistic children elevated in social anhedonia and emphasize the need for a comprehensive assessment of these symptoms in autistic children.
